# Effect of the AM Fungus *Sieverdingia tortuosa* on Common Vetch Responses to an Anthracnose Pathogen

**DOI:** 10.3389/fmicb.2020.542623

**Published:** 2020-12-18

**Authors:** Tingting Ding, Weizhen Zhang, Yingde Li, Tingyu Duan

**Affiliations:** ^1^Key Laboratory of Grassland Livestock Industry Innovation, Ministry of Agriculture and Rural Affairs, Lanzhou University, Lanzhou, China; ^2^College of Pastoral Agriculture Science and Technology, Lanzhou University, Lanzhou, China; ^3^Engineering Research Center of Grassland Industry, Ministry of Education, Lanzhou University, Lanzhou, China

**Keywords:** common vetch, disease, RNA-seq, *Colletotrichum lentis*, *Sieverdingia tortuosa*

## Abstract

*Colletotrichum lentis* Damm causes anthracnose in *Vicia sativa* L, otherwise known as common vetch. It was first reported in China in 2019. This study evaluates the effects of the arbuscular mycorrhizal (AM) fungus *Sieverdingia tortuosa* (N.C. Schenck & G.S. Sm.) Błaszk., Niezgoda, & B.T. Goto on growth and disease severity in common vetch. Our main finding is that the AM fungus increased root biomass and reduced anthracnose severity of common vetch. Responses correlated with defense, such as chitinase activity, polyphenol oxidase (PPO) activity, the concentrations of jasmonic acid and proline, and the expression of resistance-related genes (e.g., upregulated “signal transduction,” “MAPK signaling pathway,” “chitinase activity,” “response to stress,” and the KEGG pathways “phenylpropanoid biosynthesis,” “MAPK signaling pathways,” and “plant-pathogen interactions”), were also affected These findings provide insight into the mechanism by which this AM fungus regulates the defense response of common vetch to *C. lentis*.

## Introduction

Common vetch (*Vicia sativa*) is an important leguminous forage and green manure crop that is widely grown in regions with a Mediterranean climate, such as western Asia and northern Africa (Robertson et al., 1996). Common vetch grows quickly with high protein content and good palatability, which is easily digested by livestock (Akhtar and Hussain, 2009; Li and Chen, 2012). In addition, it is also an important green manure crop for its strong adaptability and ability to fix nitrogen (Cakmakci et al., 2006).

Disease is an important factor that affects the growth and production of common vetch. By the end of 2015, a total of 14 fungal pathogens, distributed in 28 countries and regions, had been reported as causing vetch diseases worldwide (Xu and Li, 2016). Among these, anthracnose is the main cause of the decline in quality and yield of common vetch. There are six pathogenic fungi known to cause anthracnose in common vetch: *Colletotrichum vicia* (Dearness, 1926), *C. villosum* (Weimer, 1945), *C. sativum* (Horn, 1952), *C. vicia-sativa* (Sawada, 1959), *C. lentis* (Xu and Li, 2015), and *C. spinaciae* (Wang Q. et al., 2019). Common vetch anthracnose caused by *C. lentis* reduced the number of effective nodules by 42.25% (Ding et al., 2019). Thus, it is important to conduct research on methods that could reduce the impact of anthracnose on common vetch.

Arbuscular mycorrhizal (AM) fungi are widely distributed in agroecosystems, and 90% of vascular plants can form mycorrhizal structures (Smith and Read, 2008). AM fungi have important functions in plants against diseases (Akhtar et al., 2011), including aboveground diseases, such as alfalfa leaf spots caused by *Phoma medicaginis* (Li et al., 2019) and powdery mildew caused by *Blumeria graminis* in barley (Ruiz-Lozano et al., 1999), as well as belowground diseases, such as pea (*Pisum sativum*) root rot caused by the *Aphanomyces euteiches* (Thygesen et al., 2004). Anthracnose of common vetch is a pathogen that can infect both the below- and aboveground organs of a host plant. Current research on the regulation of anthracnose by AM fungi is primarily concentrated in plants, such as cucumber (*Cucumis sativus*; Chandanie et al., 2006), strawberry (*Fragaria* × *ananassa*; Li et al., 2006), and cyclamen (*Cyclamen persicum*; Maya and Matsubara, 2013).

The role of AM fungi in the regulation of plant–pathogen relationships is very complex. It involves physiological, biochemical, molecular, and other mechanisms. Research by Gao et al. (2018) showed that AM fungi could affect the response of alfalfa to *Phoma medicaginis* by increasing the uptake of phosphorus and nitrogen, concentrations of chitinase, and plant growth. AM fungi can also affect plant hormones, such as salicylic acid (SA), jasmonic acid (JA), and ethylene, and signaling pathways, which are significant in plant disease resistance (Luo et al., 2013). The increased contents of SA, JA, and abscisic acid (ABA) help to enhance the resistance of plant cell walls. Moreover, SA, JA, and other signaling substances can also induce the expression of defense genes (Pozo et al., 2002), the synthesis of resistance-related proteins, and the improvement of plant stress resistance (Zhang et al., 2018).

A previous study has shown that AM fungi usually improve antioxidant enzymes related to plant defense, such as superoxide dismutase (SOD) and peroxidase (POD), when plants are exposed to pathogen stress. SOD and POD could prevent the formation of reactive oxygen species by removing reactive oxygen species and other peroxide free radicals from the plant. They could also prevent pathogen damage to the cell membrane system (Huang et al., 2015). AM fungi also decrease the content of plant malondialdehyde (MDA), one of the main products of cell membrane lipid peroxidation, the content of which can represent the degree of cell membrane damage. Generally, the more severe the damage to the plant cell membrane, the greater the amount of MDA deposited in the plant (Wang X. Y. et al., 2019). Proline also accumulates in plants when disease occurs (Mohanty and Sridhar, 1982). Few studies have been published that describe the mechanism of AM fungi in regulating anthracnose in legume forage species, and studies on the molecular mechanism revealed by transcriptome analysis (RNA-seq) are particularly lacking.

The molecular mechanism of the regulation of plant diseases by AM fungi includes promoting the expression of nutrient transporter genes and improving nutrient absorption and utilization, effectively alleviating nutrient deficiency in the plant rhizosphere (Gómez-Ariza et al., 2009; Zhang et al., 2018). Transcriptome analysis is widely used to explore plant–pathogen interactions (Huang et al., 2017). Previous studies using RNA-seq have found that *Rhizophagus intraradices* enhances the resistance of alfalfa to *P. medicaginis*. These studies initially clarified the molecular mechanism of alfalfa exposed to AM fungus to improve tolerance to disease, including phenylpropanoid biosynthesis, glutathione and phenylalanine metabolism, and other resistance- related genes (Li et al., 2019).

The common vetch, *Vicia sativa* cv. Lanjian No. 3, is a new cultivar bred by Lanzhou University, Lanzhou, China. This cultivar is a major type of green manure and legume forage crop grown in the Tibet Plateau of China and can be harvested for seeds in this area, which has an altitude of more than 4,300 m. Anthracnose is a new disease of this variety that causes severe damage to common vetch growth and production. There is currently no effective method to control this disease. In addition, there is little research focus on the interactions among pathogens, green manure crops, and AM fungi, particularly the analysis of plant defense responses using RNA-seq. Therefore, it is important to find an effective method to control the disease, particularly one that is environmentally friendly. One possibility is the application of AM fungi. A pot experiment was designed to evaluate the effects of an AM fungus on the severity of common vetch anthracnose caused by *C. lentis* using RNA-seq. We hypothesize that (i) the AM fungi will improve plant growth and general health, and (ii) the AM fungi will increase the activity of enzymes and gene expression involved in pathogen defense, thus decreasing the severity of anthracnose.

## Materials and Methods

### Plant and Fungal Materials

The seeds used in this study were common vetch (*Vicia sativa* cv. Lanjian No. 3), supplied by Forage and Turfgrass Seed Testing Center, Ministry of Agriculture and Rural Affairs, Lanzhou, China. The pathogen, *Colletotrichum lentis*, was isolated from diseased common vetch in Qingyang, Gansu Province, China. The AM fungus used in the experiments was *Sieverdingia tortuosa* (N.C. Schenck & G.S. Sm.) Błaszk., Niezgoda & B.T. Goto, previously *Glomus tortuosum* (Błaszkowski et al., 2019) and was provided by the Bank of Glomeromycota in China (Beijing). The inoculum of *S. tortuosa* was cultured in a pot of mature white clover (*Trifolium repens*). A total of 20 *g* of inoculum with 100 spores g^–1^ were added to each pot for mycorrhizal treatment (AM), and then 20 mL of sterilized water was added to each of the pots. For the non-inoculated mycorrhizal control plants’ (NM) treatments, 20 *g* of sterilized inoculum was added with 20 mL solution of the inoculum that contained microbes excluding the AM fungus, which was added to the soil with a previously prepared microbial filtrate (50 μm) as described by Gao et al. (2018).

### Growth Medium

We obtained the soil from a field in which common vetch was grown, and it was sterilized in an autoclave at 121°C for 2 h. We sieved the sand through a 2 mm sieve and sterilized it in an oven at 180°C for 24 h. The medium was composed of a 1:3 mix of soil and sand; the total *N* was 15.78 mg⋅kg^–1^. [The total *P* was 18.11 mg⋅kg^–1^, and the pH was 7.1.]. Before transplanting the seedlings, 20 mL solution of the non-sterilized field soil that contained microbes with the exception of the AM fungus was added to the growth medium as a microbial filtrate (50 μm).

### Experimental Design

A crossed, two-factor experiment was designed as follows: AM fungus (two treatments: inoculated; uninoculated) × *C. lentis* (two treatments: inoculated, uninoculated) = a total of four treatments (NM, NMP, AM, and AMP). Each treatment was conducted with six pots. Therefore, a total of 24 pots were planted in this experiment. *C. lentis* was inoculated onto half of each AM and NM treatment 37 days after the emergence of common vetch.

Common vetch seeds were surface-sterilized with 10% H_2_O_2_ for 5 min and rinsed three times with sterilized water. The sterilized seeds were evenly placed in a petri dish and left in the dark at 25°C for 24 h. Six seeds were planted in each pot, and the plants were thinned to four plants per pot after 7 days of growth. After 37 days of growth in a greenhouse, the pathogens were inoculated as described by Li et al. (2019) with some modifications. Specifically, the *C. lentis* suspension contained ∼108 CFU⋅mL^–1^ conidia, with two drops of Tween-20 added to the conidial suspension for inoculation. The suspension (20 mL per pot) was sprayed on the plant, which had been wounded on the leaves and stems with insect needles. The plant was then covered with a black plastic bag for 48 h to retain moisture. Plants uninoculated with pathogens were wounded as described and sprayed with sterile water instead of the spore suspension.

The experiment was conducted in a greenhouse (23–28°C day/20–25°C night) with a radiation range of 480–850 mmol/m^2^⋅s during the growth period (day 14 h/night 10 h). The plants were watered with tap water to ensure that the soil moisture was maintained at 60% of the maximum field water capacity. Each week, 100 mL of modified Hoagland nutrient solution (without phosphorus) was added to the pots. Growth was monitored regularly throughout the experiment by counting the number of leaves on each plant, measuring its height, and counting the number of branches on each plant. The disease incidence was recorded every 3 days (Gao et al., 2018). The disease index was recorded, and the plants were harvested 18 days after inoculation with the pathogen. The disease index was recorded by visual inspection of the leaf grade by percentage of lesion area to leaf area and divided into the following six levels: 0, no disease spots; 1, ≤5% of the total leaf area covered with necrotic spots; 2, 6–20%; 3, 21–50%; 4, 51–75%; and 5, >75%. Disease index = 100 × [Σ (number of diseased leaves × level of disease)/(total number of leaves × 5)].

### Plant Harvest and Measurement

At harvest, fresh shoots of the plant were divided into eight parts, and 0.2 *g* was randomly selected and stored at −80°C in liquid nitrogen for subsequent RNA-seq analysis. Then, 0.2 *g* of shoots were sampled to measure the activities of SOD, polyphenol oxidase (PPO), and POD as previously described (Li, 2000). Five approximately 0.2 *g* subsamples of shoots were used to detect chitinase activity (Yang et al., 2011), JA (Wang, 2006), SA (El-Beltagi et al., 2017), MDA (Wang, 2006), and proline (Zhang et al., 1990), respectively. In addition, 0.5 *g* of shoots was used to re-isolate the pathogen *C. lentis* to meet Koch’s postulates (Li et al., 2019). The remaining portion of the shoots was used to determine the dry weight (from the fresh weight: dry weight ratios of the subsamples). The AM colonization and colonization intensity, disease incidence, disease index, proline, and MDA content were measured using six pots of each treatment. The assays of PPO, POD, SOD, and chitinase, the contents of JA and SA, and transcriptome sequencing used three replicates that were randomly selected from six replicates of each treatment.

The plant roots were carefully washed to determine the root area using a root scanner (Expression 11000XL, Epson, Beijing, China). Approximately 0.2 *g* of roots was used to determine the extent of AM colonization (Giovannetti and Mosse, 1980). The remaining portion of the collected roots was used to determine the dry weight (based on total fresh weight and sample dry weight).

### Transcriptome Sequencing

#### RNA Extraction

Approximately 0.2 *g* of plant leaves were randomly sampled, added to cryotubes, quickly frozen in liquid nitrogen, and stored at −80°C. TRIzol Reagent (ThermoFisher Scientific, Waltham, MA, United States) was used to extract total RNA from the leaf tissue. To remove the DNA, RNA samples were treated with DNase I. A Qubit RNA assay kit (ThermoFisher Scientific) was used to determine the purity of the total RNA samples, and their integrity was assessed on an Agilent Bioanalyzer 2100 system (Agilent Technologies, Carpinteria, CA, United States).

#### cDNA Library Construction and Sequencing

Total RNA was extracted for cDNA synthesis. The cDNA library was constructed using an NEBNext Ultra^TM^ RNA Library Preparation Kit (NEB, Beverly, MA, United States). The cDNA library was sequenced using a HiSeq X Reagent Kit on a Hi-seq X-10 (Illumina, San Diego, CA, United States), according to the RNA-seq instructions, to generate an 150 bp paired end original reading.

#### Analysis of the RNA-seq Data

RNA-seq data analysis was conducted by filtering out low quality reads. Trinity was used for the *de novo* assembly of clean reads, and then Tgicl was used to cluster and reduce the assembled transcripts twice to finally obtain All-Unigenes. BUSCO Bowtie2 was used to compare clean reads to the reference gene sequence. RSEM was then used to calculate the levels of expression of the genes and transcripts. Unigenes were then functionally annotated (KEGG, GO, and NR) after each sample in All-Unigene. The expression levels were calculated based on the genes that were differentially expressed between the different treatments. Fragments Per Kilobase per Million (FPKM) was used to describe the level of gene expression (Trapnell et al., 2010).

#### Analysis of Differentially Expressed Gene

The DESeq package was used to identify the Differentially Expressed Gene (DEGs; Wang et al., 2010). Significant differences in gene expression were determined using the FDR threshold < 0.05 and the absolute value of log2 (fold change) ≥ 1 (Zhuang et al., 2018). To determine the primary biological functions and pathways of DEGs, the R function phyper were used for the Gene Ontology (GO) database and Kyoto Encyclopedia of Genes and Genomes (KEGG) enrichment analyses (Fu et al., 2018). GO analysis was mapped using the R 3.5.2 “ggplot2” package and was performed on all the DEGs. The DEGs were annotated in the GO database and assigned to three main categories. Gene expression calorimetry maps were generated using the R 3.5.2 “Pheatmap” package.

#### Real-Time Quantitative Polymerase Chain Reaction Validation

To verify the results of the transcriptome analysis, Quantitative Polymerase Chain Reaction (qRT-PCR) was used to measure the levels of expression of eight selected genes related to plant defense. Each gene verification was conducted using three independent biological replicates. cDNA was synthesized from the extracted total RNA using the TUREscript 1st Stand cDNA SYNTHESIS kit (Aidlab, Shanghai, China). qTOWER 2.0/2.2 quantitative/real-time PCR thermocycler (Germany) was used to perform the qRT-PCR assays. The qRT-PCR reaction system consisted of 5 μL of 2XYBR^®^ Green Supermix, 1 μL of cDNA, 0.5 μL of primer, and 3 μL of ddH_2_O in a total volume of 10 μL. Then, qRT-PCR was performed as follows: 95°C, 3 min; 95°C, 10 s; 58°C, 30 s, 39 cycles, followed by a melting curve (60°C to 95°C, +1°C per cycle, holding time 4 s). The qRT-PCR was conducted in triplicate to eliminate effects such as the noise from machine equipment, and the 2^–ΔΔ*C*^_*T*_ method was used to calculate the relative expression level of the genes (Livak and Schmittgen, 2001). qPCR was mapped using Microsoft Excel (Redmond, WA, United States).

#### Statistical Analysis

Data were presented as the means ± standard errors of six replicates, with the exception that the transcriptome sequencing was conducted using three replicates. The data were analyzed using an analysis of variance (ANOVA) with JMP 4 statistical software (SAS Institute, Inc., Cary, NC, United States) at the 0.05 probability level. Data for the percent of AM colonization were ARCSIN-transformed to achieve normality.

## Results

### AM Fungus Colonization

**FIGURE 1:**
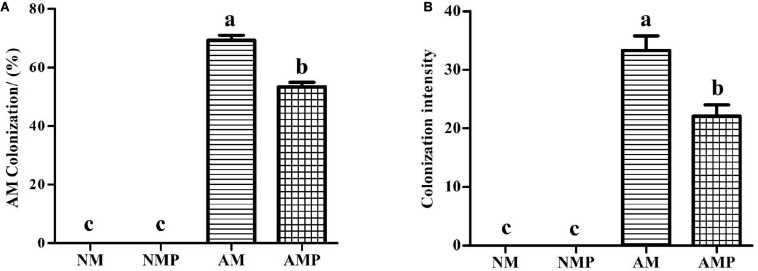
AM colonization **(A)** and colonization intensity **(B)** in the roots of common vetch. NM = uninoculated with *S. tortuosa*, AM = inoculated with *S. tortuosa*, NMP = NM inoculated with *C. lentis*, and AMP = AM inoculated with *C. lentis*. Different lowercase letters on the bars means there is significant difference across treatments at *P* < 0.05 by Tukey’s HSD test.

No mycorrhizal structure was detected in the NM treatment (Figure 1). The percentage of AM colonization and colonization intensity in the roots of *V. sativa* were 69.17% and 33.32% in the AM treatment, respectively. Infection with *C. lentis* reduced the percentage and intensity of AM colonization by 22.9% and 33.73% (*P* < 0.05), respectively.

### Assessment of Disease Severity

**FIGURE 2:**
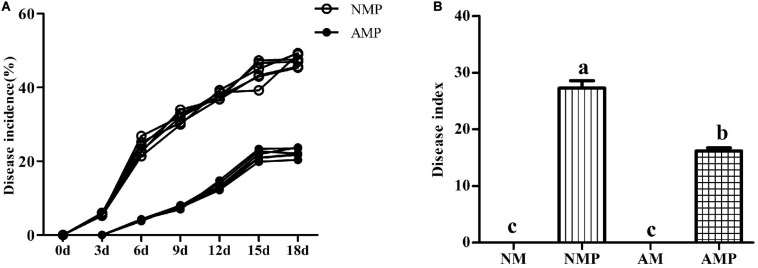
Disease incidence **(A)** and disease index **(B)** of common vetch anthracnose under arbuscular mycorrhizal and disease treatments. NM = uninoculated with *S. tortuosa*, AM = inoculated with *S. tortuosa*, NMP = NM inoculated with *C. lentis*, and AMP = AM inoculated with *C. lentis*. Different lowercase letters on the bars means there is significant difference across treatments at *P* < 0.05 by Tukey’s HSD test.

At the third day of pathogen inoculation, NM plants showed typical necrotic spots, whereas necrotic spots occurred on the sixth day in AM plants (Figure 2). The disease incidence of the NM treatment was 23.82% higher than that of the AM treatment. Subsequently, the disease incidence in both the NM and AM treatments continued to increase, reaching 47.30% and 19.77%, respectively, on the 18th day of the pathogen inoculation. The disease incidence of the AM treatment was always lower than that of the NM treatment. The disease incidence and disease index of the NM treatment were 53% and 40.77% (*P* < 0.05) higher than that of the AM treatment on the 18th day after inoculation, respectively.

### Plant Growth

Colonization with the AM fungus significantly increased the height of common vetch shoots and the length of common vetch roots. In particular, the root biomass and height of AM plants were 87.79% and 58.08% higher than that NM plants, respectively. The AM fungi increased the length of the shoots. However, there were fewer branches of the plant than those of the NM plants. Thus, the biomass of the AM and NM plants was the same. In addition, the root length and root area of the AM plants were 42.32% and 71.96% greater than that of NM plants, respectively, (*P* < 0.05). Pathogen infection had no significant influence on plant growth owing to the short period of the infection of pathogen (Table 1).

**TABLE 1:** Effects of AM fungus *S. tortuosa* and *C. lentis* on the growth of common vetch.

Treatment	Shoot biomass(g/plant)	Root biomass(g/plant)	Plant height (cm)	Root length (cm)	Root area (cm^2^)
NM	5.83 ± 0.09^*a*^	1.72 ± 0.15^*b*^	9.18 ± 0.07^*b*^	12.17 ± 0.05^*b*^	20.08 ± 0.13^*b*^
AM	6.09 ± 0.09^*a*^	3.23 ± 0.54^*a*^	14.51 ± 0.11^*a*^	17.32 ± 0.23^*a*^	34.53 ± 0.17^*a*^
NMP	5.92 ± 0.17^*a*^	1.37 ± 0.31^*b*^	9.33 ± 0.13^*b*^	11.91 ± 0.11^*b*^	22.31 ± 0.08^*b*^
AMP	5.98 ± 0.23^*a*^	2.91 ± 0.37^*a*^	13.26 ± 0.07^*a*^	17.66 ± 0.37^*a*^	34.22 ± 0.29^*a*^

*Note: NM = uninoculated with *S. tortuosa*, AM = inoculated with *S. tortuosa*, NMP = NM inoculated with *C. lentis*, and AMP = AM inoculated with *C. lentis*, Different lowercase letters on the bars means there is significant difference across treatments at *P* < 0.05 by Tukey’s HSD test.*

### Plant Enzyme Activities and Chemical Contents

**FIGURE 3:**
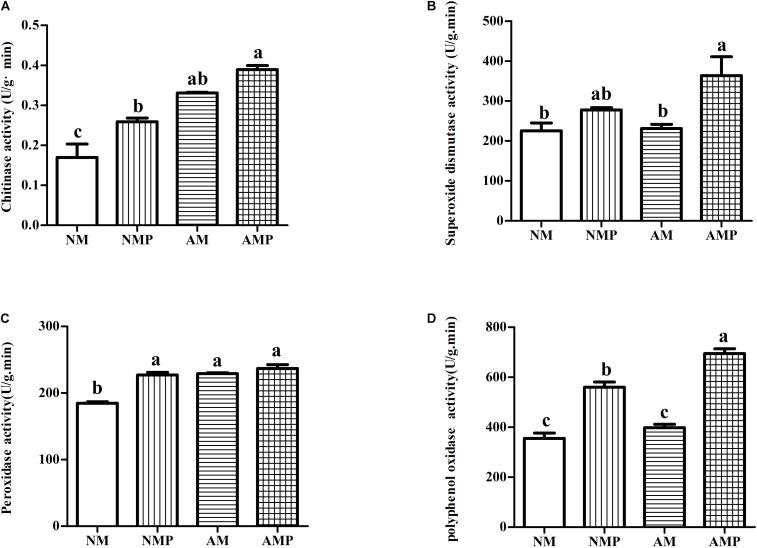
Chitinase **(A)**, superoxide dismutase **(B)**, peroxidase **(C)**, and polyphenol oxidase **(D)** activity of common vetch under arbuscular mycorrhizal and disease treatments. NM = uninoculated with *S. tortuosa*, AM = inoculated with *S. tortuosa*, NMP = NM inoculated with *C. lentis*, and AMP = AM inoculated with *C. lentis*. Different lowercase letters on the bars means there is significant difference across treatments at *P* < 0.05 by Tukey’s HSD test.

Superoxide dismutase and PPO are important antioxidant enzymes in plants. Common vetch that had been colonized with AM fungus presented higher chitinase, SOD, POD, and PPO activities than those of the NM (Figure 3). The chitinase and POD activities of plants in the AM treatment increased by 95.21% (*P* < 0.05) and 24.15% (*P* < 0.05), respectively. Chitinase, POD, and PPO of plants in the AMP were 52.64% (*P* < 0.05), 23.10% (*P* < 0.05), and 36.55% (*P* < 0.05) higher than that of the control, respectively.

**FIGURE 4:**
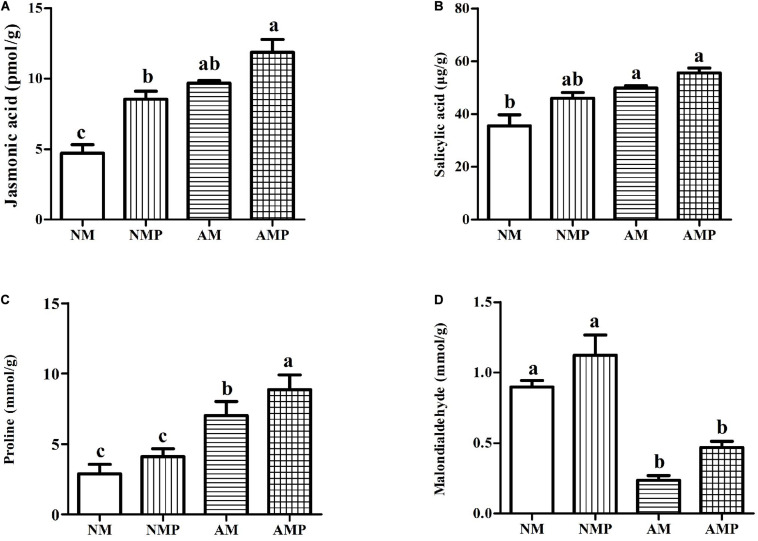
Jasmonic acid **(A)**, salicylic acid **(B)**, proline **(C)**, and malondialdehyde **(D)** contents under arbuscular mycorrhizal and disease treatments. NM = uninoculated with *S. tortuosa*, AM = inoculated with *S. tortuosa*, NMP = NM inoculated with *C. lentis*, and AMP = AM inoculated with *C. lentis*. Different lowercase letters on the bars means there is significant difference across treatments at *P* < 0.05 by Tukey’s HSD test.

The AM fungus exhibit increased the contents of JA, SA, and proline in healthy plants by 81.72, 56.39, and 143.44% (*P* < 0.05) and significantly decreased the content of MDA by 73.68% (*P* < 0.05) when they were compared with the NM treatment. Pathogen infection and treatment with the AM fungus (AMP) resulted in the highest levels of plant defense-related materials. Common vetch infected with the pathogen (AMP and NMP) had relatively higher contents of JA, SA, proline, and MDA (Figure 4). The combined AM and pathogen treatment significantly increased the levels of JA and proline in the plant by 38.84% and 115.57% (*P* < 0.05), respectively, and significantly decreased the content of MDA by 58.27% (*P* < 0.05) compared with AM alone. These compounds are closely related to the resistance of plants to pathogens, and their increase is consistent with the higher activities of SOD, POD, and PPO.

### RNA-seq and Mapping

After removing the low-quality sequences, joint contamination sequences, and reads with unknown base N content sequences, a total of 132.65 Gb of data was obtained using the BGISEQ-500 platform. An average of 74.62 Mb, 73.24 Mb, 74.18 Mb, and 72.72 Mb clean reads from NM, AM, NMP, and AMP, respectively, were obtained. The percentage of GC content in the treatments was similar (39.62%). The Q20 and Q30 percentages were greater than 97.19% and 89.11%, respectively. Using Bowtie2, 18.3–21.15% of the clean reads were mapped to the reference genome (Supplementary Table 1).

The length distribution of the unigenes is shown in Supplementary Figure 1. After assembly and redundancy, 108,035 unigenes were obtained. An average of 43,218.33, 46,833.33, 47,213, and 46,833.33 unigenes were obtained from the NM, AM, NMP, and AMP treatments, respectively. The average length of the unigenes was 1,659 bp.

### Analysis of DEGs Resulting From Different Treatments

**FIGURE 5:**
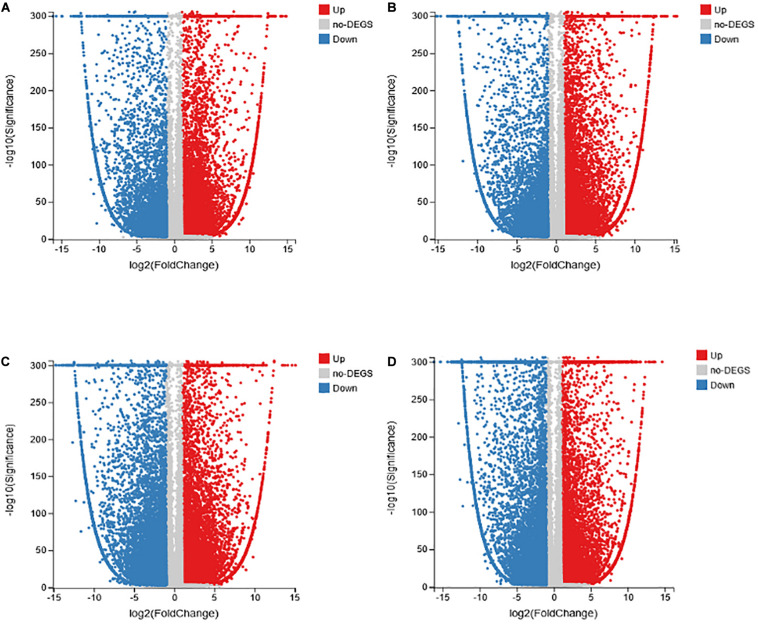
Analysis of DEGs in common vetch in NM-AM **(A)**; NM-NMP **(B)**; AM-AMP **(C)**; and NMP-AMP **(D)**. NM = uninoculated with *S. tortuosa*, AM = inoculated with *S. tortuosa*, NMP = NM inoculated with *C. lentis*, and AMP = AM inoculated with *C. lentis*. The *x*-axis represents the change in gene expression folds in different samples, the *y*-axis represents the statistical significance of the difference in gene expression changes in the sample; the red dot indicates the up-regulated gene with significant differential expression; the blue dot indicates the up-regulated gene with significant differential expression.

To explore the changes in the transcriptional level of the transcriptome of common vetch inoculated with AM fungus and anthracnose, a *q* value ≤ 0.05 and | log2 (fold change) | ≥1 was used to determine the significant difference in the level of expression of the genes in different treatments. A total of 20,886 to 34,837 DEGs were screened in the four treatment groups, with 17,577, 16,249, 13,951, and 12,671 upregulated expression in the NM-AM, NM-NMP, AM-AMP, and NMP-AMP treatments, respectively. In addition, 10,294, 10,511, 20,886, and 17,522 DEGs were downregulated in NM-AM, NM-NMP, AM-AMP, and NMP-AMP, respectively, (Figure 5).

### Differential Gene Statistics of Pathogenesis-Related Proteins

Pathogenesis-related (PR) protein transcripts accumulated in plants infected with *C. lentis*. Compared with the NM treatment, PR-1, PR-4, PR-5, PR-10, PR-bet VI, and PR-12 were significantly upregulated in the NMP treatment (Table 2). Furthermore, PR protein genes, including PR-1, PR-5, PR-10, PR-bet VI, and PR-12, were significantly upregulated in the AMP treatment compared with that of the NMP (Table 3).

**TABLE 2:** Genes regulating PR proteins in the NM vs NMP.

Gene ID	Pathogenesis-related (PR)protein	log2FoldChange	*q*-value	
*CL10565.Contig2_All*	Pathogenesis-related protein 1-like (PR-1)	6.914960844	0	Up
*CL4104.Contig1_All*	Pathogenesis-related protein 1-like (PR-1)	3.276764958	2.66E-11	Up
*Unigene16276_All*	Pathogenesis-related protein 1-like (PR-1)	2.472089136	4.67E-09	Up
*Unigene33856_All*	Pathogenesis-related protein 1-like (PR-1)	4.82001244	9.11E-05	Up
*CL7287.Contig3_All*	Chitinase (PR-4)	6.208040452	0	Up
*CL8632.Contig1_All*	Chitinase (PR-4)	3.637342745	7.49E-26	Up
*Unigene21534_All*	Pathogenesis-related thaumatin family protein (PR-5)	4.494370584	0	Up
*CL2943.Contig1_All*	Pathogenesis-related thaumatin family protein (PR-5)	1.5734712	9.27E-15	Up
*CL6172.Contig2_All*	Pathogenesis-related thaumatin family protein (PR-5)	1.652097894	1.18E-41	Up
*Unigene14727_All*	Pathogenesis-related thaumatin family protein (PR-5)	1.597620019	4.15E-13	Up
*Unigene15571_All*	Pathogenesis-related thaumatin family protein (PR-5)	2.898047044	0	Up
*CL2217.Contig10_All*	Ribonuclease-like (PR-10)	3.409959051	0	Up
*CL2217.Contig4_All*	Ribonuclease-like (PR-10)	3.165161051	1.01E-58	Up
*CL2217.Contig5_All*	Ribonuclease-like (PR-10)	3.127873382	0	Up
*CL2217.Contig6_All*	Ribonuclease-like (PR-10)	2.882958125	1.41E-55	Up
*CL2217.Contig7_All*	Ribonuclease-like (PR-10)	3.544779339	0	Up
*CL2217.Contig8_All*	Ribonuclease-like (PR-10)	4.65338736	0	Up
*CL2217.Contig9_All*	Ribonuclease-like (PR-10)	1.299242287	0	Up
*CL6512.Contig3_All*	Pathogenesis-related protein bet VI family protein	2.186973416	0	Up
*CL7966.Contig5_All*	Defensin (PR-12)	1.133964	7.41E-13	Up
*CL7966.Contig6_All*	Defensin (PR-12)	1.43156	3.19E-42	Up
*Unigene1517_All*	Defensin (PR-12)	9.488391	4.26E-64	Up

*Note: NM = uninoculated with *S. tortuosa*, NMP = NM inoculated with *C. lentis*.*

**TABLE 3:** Genes regulating PR proteins in the NMP vs AMP.

Gene ID	Pathogenesis-related (PR)protein	log2FoldChange	*q*-value	
*Unigene35748_All*	Pathogenesis-related protein 1-like (PR-1)	8.143527242	1.30E-30	Up
*Unigene16276_All*	Pathogenesis-related protein 1-like (PR-1)	5.595924142	0	Up
*CL2943.Contig3_All*	Pathogenesis-related thaumatin family protein (PR-5)	1.252382661	3.28E-80	Up
*CL6172.Contig1_All*	Pathogenesis-related thaumatin family protein (PR-5)	1.457641166	0	Up
*CL6172.Contig4_All*	Pathogenesis-related thaumatin family protein (PR-5)	2.141419579	0.000224	Up
*CL6172.Contig5_All*	Pathogenesis-related thaumatin family protein (PR-5)	2.592027224	3.33E-15	Up
*CL7746.Contig4_All*	Pathogenesis-related thaumatin family protein (PR-5)	4.274771775	1.06E-76	Up
*CL886.Contig2_All*	Pathogenesis-related thaumatin family protein (PR-5)	1.624081395	3.09E-15	Up
*Unigene14726_All*	Pathogenesis-related thaumatin family protein (PR-5)	2.806599926	1.13E-233	Up
*Unigene14727_All*	Pathogenesis-related thaumatin family protein (PR-5)	3.835274084	0	Up
*Unigene22015_All*	Pathogenesis-related thaumatin family protein (PR-5)	9.849189248	0	Up
*CL2943.Contig2_All*	Pathogenesis-related thaumatin family protein (PR-5)	1.244505428	2.27E-19	Up
*Unigene36700_All*	Pathogenesis-related thaumatin family protein (PR-5)	8.748953846	7.53E-43	Up
*Unigene19632_All*	Ribonuclease-like (PR-10)	2.101707067	2.83E-28	Up
*Unigene17887_All*	Pathogenesis-related protein bet VI family protein	3.830161161	9.21E-174	Up
*CL2213.Contig4_All*	Pathogenesis-related protein bet VI family protein	1.217626102	2.62E-13	Up
*CL7966.Contig1_All*	Defensin (PR-12)	1.088853648	3.04E-110	Up
*CL7966.Contig2_All*	Defensin (PR-12)	2.696359212	6.39E-16	Up
*CL7966.Contig3_All*	Defensin (PR-12)	3.669596054	1.24E-138	Up

*Note: NMP = inoculated with *C. lentis*, AMP = inoculated with *S. tortuosa* and *C. lentis*.*

### GO Analysis

In the NMP and AMP comparison, 3,213 GO categories were identified, of which 87 GO categories were significantly enriched (*q*-value ≤ 0.05). For the molecular function, cellular component, and biological process, 48, 8, and 31 GO terms were significantly enriched, respectively. In the NM and NMP comparison, the most significantly enriched DEGs in the three major functional categories, molecular function, cellular component, and biological process, were 24, 16, and 13, respectively. GO enrichment analysis identified 3,013 GO categories, of which 31 were significantly enriched in the NM-NMP treatment group (*q*-value ≤ 0.05). In addition, 18, 3, and 10 GO terms were significantly enriched in MF, CC, and BP, respectively.

**FIGURE 6:**
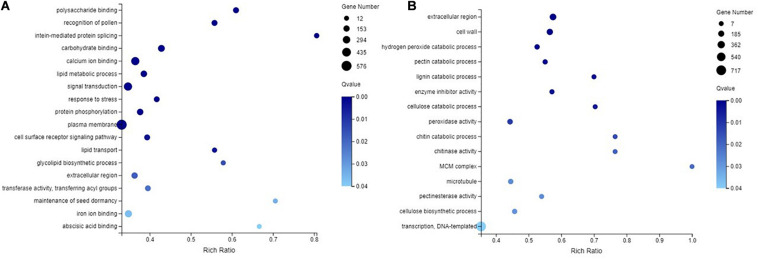
GO enrichment analysis in NM-NMP **(A)** NMP-AMP **(B)**. NM = uninoculated with *S. tortuosa*, NMP = NM inoculated with *C. lentis*.

Some GO categories that were significantly enriched (*q*-value ≤ 0.05) in the NM-NMP treatment group and NMP-AMP treatment group were selected (Figure 6). In the NM and NMP comparison, a total of 576, 163, 373, 103, 14, and 137 genes were enriched for plasma membrane (GO:0005886, *q*-value = 0.000565246), extracellular region (GO:0005576, *q*-value = 0.01765339), signal transduction (GO:0007165, *q*-value = 0.000113079), response to stress (GO:0006950, *q*-value = 0.000439065), ABA binding (GO:0010427, *q*-value = 0.03836736), and calmodulin binding (GO:0005516, *q*-value = 9.15E-06; Figure 6A). In the NMP and AMP comparison, a total of 7, 21, 13, 19, 40, 13, and 34 genes were enriched in the MCM complex (GO:0042555, *q*-value = 0.01763115), lignin catabolic process (GO:0046274, *q*-value = 0.002234576), chitin catabolic process (GO:0006032, *q*-value = 0.0125329), cellulose catabolic process (GO:0030245, *q*-value = 0.003835744), enzyme inhibitor activity (GO:0004857, *q*-value = 0.002680624), chitinase activity (GO:0004568, *q*-value = 0.01604237), and pectinesterase activity (GO:0030599, *q*-value = 0.022718), respectively. The expression of all the genes enriched in the MCM complex and lignin catabolic process were significantly upregulated (Figure 6B).

### KEGG Analysis

**FIGURE 7:**
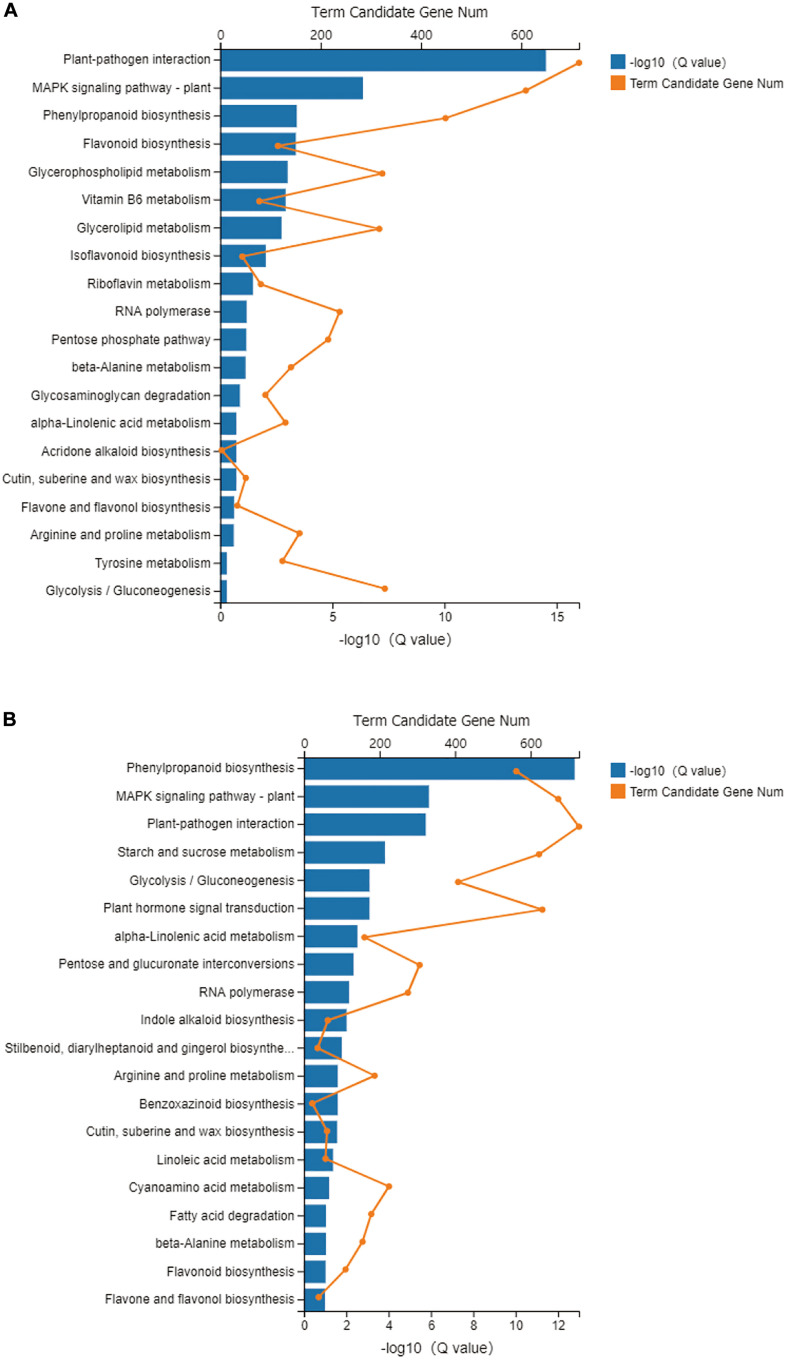
KEGG pathway enrichment analyses in NM-NMP **(A)** NMP-AMP **(B)**. NM = uninoculated with *S. tortuosa*, AM = inoculated with *S. tortuosa*, NMP = NM inoculated with *C. lentis*, and AMP = AM inoculated with *C. lentis*.

Using KEGG pathway annotation, all the metabolic pathways in each treatment were analyzed. In the NM and NMP comparison, 10,590 DEGs were annotated, accounting for 39.57% of the total number of DEGs. These DEGs were enriched in 133 metabolic pathways. The most significant metabolic pathways for the enrichment of DEGs were selected (*q*-value < 0.05; Figure 7A), namely, plant-pathogen interaction (ko04626), mitogen-activated protein kinase (MAPK) signaling pathway-plant (ko04016), phenylpropanoid biosynthesis (ko00940), flavonoid biosynthesis (ko00941), glycerophospholipid metabolism (ko00564), vitamin B6 metabolism (ko00750), glycerolipid metabolism (ko00561), isoflavonoid biosynthesis (ko00943), and riboflavin metabolism (ko00740). In the NMP and AMP comparison, 11,948 DEGs were annotated into 133 specific metabolic pathways, accounting for 39.57% of the total number of DEGs. The top 10 metabolic pathways with the most significant enrichment of DEGs were screened (Figure 7B; *q*-value < 0.05), resulting in phenylpropanoid biosynthesis (ko00940), MAPK signaling pathway – plant (ko04016), plant-pathogen interaction (ko04626), starch and sucrose metabolism (ko00500), glycolysis/gluconeogenesis (ko00010), plant hormone signal transduction (ko04075), alpha-Linolenic acid metabolism (ko00592), Pentose and glucuronate interconversions (ko00040), RNA polymerase (ko03020), and indole alkaloid biosynthesis (ko00901).

### Transcript Levels of the Genes Related to Phenylpropanoid Biosynthesis

**FIGURE 8:**
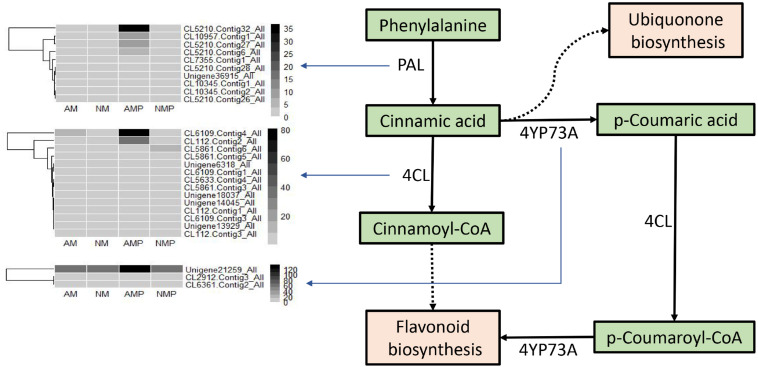
Regulating genes expression and graphical overview of the phenylpropane biosynthesis. NM = uninoculated with *S. tortuosa*, AM = inoculated with *S. tortuosa*, NMP = NM inoculated with *C. lentis*, and AMP = AM inoculated with *C. lentis*.

Transcriptional expression profiles of the genes that control the phenylpropanoid biosynthetic pathway is illustrated in Figure 8. The results obtained in this study indicate that the levels of expression of *CL5210.Contig32_All* (phenylalanine ammonia-lyase-like protein), *CL10957.Contig1_All* (dirigent protein 22-like), *CL5210.Contig27_All* (phenylalanine ammonia-lyase-like protein), and *CL5210.Contig6_All* (hypothetical protein TSUD_337780) in the AMP treatment were higher than those in the other treatments during the process of phenylalanine synthesis of cinnamic acid. The levels of expression of *CL6109.Contig4_All* (CoA ligase-like protein) and *CL112.Contig2_All* (CoA ligase-like protein) in the AMP treatment were higher than other treatments during the process of cinnamic acid synthesis, cinnamoyl-CoA and *p*-coumaric acid, and the synthesis of *p*-cinnamoyl-CoA. During the process of cinnamic acid synthesis, *p*-coumaric acid, and *p*-cinnamoyl-CoA to flavonoid biosynthesis, the expression of *Unigene31259_All* (glycoside hydrolase family 1 protein) in the AMP treatment were higher than that in the other treatments.

### qRT-PCR

Eight DEGs were randomly selected for qRT-PCR validation to verify the reliability of DEGs in transcriptome sequencing results (Supplementary Figure 2 and Supplementary Table 2). The results showed that there were *Unigene10226_All*, *Unigene16284_All*, *CL672.Contig2_All*, *CL7746.Contig4_All*, and *CL9889.Contig3_All* DEGs. These five DEGs were consistent with those of transcriptome (RNA-seq) sequencing. The expression levels of *CL672.Contig2_All* and *CL7746.Contig4_All* were the same in the RNA-seq and qRT-PCR experiment. The level of expression of *Unigene10226_All* and *CL9889.Contig3_All* in RNA-seq and qRT-PCR were the highest in the treatment inoculated with *S. tortuosa* and *C. lentis*. RNA-seq and qRT-PCR of *Unigene16284_All* were the highest in the treatment inoculated with *S. tortuosa*.

The remaining genes were inconsistent in both the RNA-seq and qRT-PCR analyses, which is because some genes have a particularly complex transcript format, whereas in RNA-seq, different transcripts are routinely combined into one transcript. In the qRT-PCR validation, if the primers were not designed in the transcript shared exon of the gene, only some specific transcripts were covered, resulting in a lack of sensitivity and discrimination in the primers and inaccurate expression of the qPCR. In this study, half of the DEGs were found to be very similar in the qRT-PCR and RNA-seq analyses, indicating that RNA-seq sequencing data were highly reliable.

## Discussion

In this study, transcriptome analysis (RNA-seq) was used to reveal the effects of AM fungus on anthracnose in common vetch caused by *C. lentis*. The colonization of AM fungus improved the growth of plant roots with changed morphology of the aboveground part of the plants. It further increased plant defense enzyme activity, such as chitinase and PPO, enhanced disease-related protein gene expression, and reduced the concentration of MDA, to improve the resistance of the plant to *C. lentis*. Relevant defense genes, such as those encoding chitinase, were upregulated in plants inoculated with *S. tortuosa*. Our hypothesis that the AM fungus improves plant growth and decreases the occurrence of common vetch anthracnose was confirmed.

Mycorrhizal structures were observed in the roots of AM treatments, but no mycorrhizal structures were observed in the non-inoculated roots, which indicated that the inoculation of AM fungus was successful. Pathogen infection significantly reduced the percentage of AM colonization and colonization intensity. A previous study showed that the disease can cause the blockage of the vascular system, resulting in xylem pressure that increases the hydraulic resistance of the vascular system and reduces the photosynthesis rate, thereby reducing the supply of photosynthetic products to the roots (Corriveau and Carroll, 1984). Disease can also reduce the expression of genes related to photosynthesis (Li et al., 2019), thus reducing the C provided to the AM fungus. The reasons described above may have caused the decline of AM fungus after infection with *C. lentis*. The colonization with AM fungus resulted in a delay in the onset of disease, and the incidence and disease index were reduced in plants during the experiment. This evidence indicated that there was competition between the AM fungus and pathogen at the beginning of infection. The inhibitory effects of the AM fungus to the pathogen were visible in the growth of the plant. The reduction in the disease incidence and disease index was consistent with previous studies on alfalfa leaf spot caused by *P. medicaginis* (Li et al., 2019). Treatment with the AM fungus can delay the occurrence of plant diseases and reduce the disease incidence and disease index. The AM fungus improved the root growth of plants, resulting in overall “healthier” plants (Table 1). This may be another reason why the AM fungus increased resistance to the pathogen (Gao et al., 2018). In addition, infection by the pathogen did not decrease the biomass of plant. The reason is that the disease primarily causes leaves to fall in the field during the later growth period, thus, causing the yield loss of biomass. However, fallen leaves were not observed owing to the short time in our greenhouse pots experiment. The plants were more sensitive to pathogen stress at the occurrence of early stages of the disease.

Chitinase can decompose chitin exposed to the tip of fungal hyphae and directly participates in plant disease resistance (Selitrennikoff, 2001). This study found that the chitinase activity of the plant was significantly increased after the plants were infected by the pathogen. The AM fungus significantly increased the amount of chitinase activity in the diseased plants. The results show that, when infected by the pathogen, the increase in chitinase activity is an important way for plants to resist pathogens. The AM fungus promotes the ability of plants to be resistant to pathogens by regulating chitinase activity. An increase in the content of proline was conducive to protecting the transformation and destruction of proteins, increasing the activity of proteases, and strengthening plant resistance to stress (Zhu et al., 2009). Our research found that the AM fungus significantly reduced the content of MDA of plants and increased the content of proline, thus reducing the degree of disease stress in common vetch. PPO covalently regulates nucleophilic amino acids through quinones to form an anti-nutritional mechanism, allowing plants to directly resist pathogens (Duffey and Felton, 1991). In this study, pathogens significantly increased PPO activity in all the plants, and the PPO activity of the AMP treatment was significantly higher than that of the NMP treatment, indicating that the AM fungus may enhance the resistance of plants to pathogens by increasing the activity of PPO in the plants. POD and SOD are plant antioxidant enzymes (Li et al., 2019). We found that pathogens significantly increased the activities of POD and SOD in plants, suggesting that plants may improve their defense to pathogens by enhancing the activities of these enzymes. However, the ability of antioxidant enzymes to scavenge active oxygen free radicals against disease is limited. It is not the only way for plants to resist pathogens; it can be combined with other ways to resist pathogen infection (Xia et al., 2015; Wang X. Y. et al., 2019). Several plant hormones are known to mediate plant defense responses against pathogens (Verma et al., 2016), of which the increase in concentration of SA and JA helps to enhance the activities of POD and PPO (Moosa et al., 2019). The results showed that inoculation with the AM fungus increased the concentrations of JA and SA in plants infected with pathogens. This supports the results that AM fungi increase PPO activity in diseased plants. In addition, both JA and SA can induce plant resistance and specific gene expression, synthesize related defense proteins, such as PAL and PR-1, and promote plants to produce defense-related substances, such as lignin, thereby improving plant disease resistance (Wang et al., 2000; Wasternack and Hause, 2002). Increasing the expression of genes related to disease resistance hormones in plants is a response of plants to a pathogen.

PR proteins usually accumulate when plants are exposed to pathogens (Liu and Ekramoddoullah, 2006). The production and accumulation of PR proteins play an important role in the resistance of plants to biological stress (Wen et al., 2008). The results of the transcriptome analysis showed that the AM fungus enhanced the expression of genes for PR proteins. PR proteins in diseased plants, including PR-1, PR-4, PR-5, PR-10, and PR-12, were significantly upregulated in the common vetch infected with *C. lentis*. PR-5 and PR-12 also have antifungal activity (Lay and Anderson, 2005; Misra et al., 2016). The biological activity of PR-1 is uncertain, but it may be induced by pathogens to enhance the plant’s defensive state (Loon and Strien, 1999). PR-4 and PR-10 are important in defense against pathogens (Park et al., 2003; Caporale et al., 2004). The expression of genes that regulate PR proteins was improved in the NMP and AMP treatments, indicating that pathogens may induce the defense system of a plant. Our results showed that the AM fungus improved the resistance of plants to pathogens by regulating PR proteins, since the expression of their genes was upregulated. It was worth noting that JA and SA can induce PR protein-related gene expression in plants (Irigoyen et al., 2020). The upregulation of the genes for PR proteins in AM plants is linked with the higher concentrations of JA and SA in the plant shoots.

The GO analysis found eight up-regulated DEGs that encoded chitinase activity in the NMP-AMP treatment. This resulted in the rich GO term “chitinase activity” (GO: 0004568), which was not found in the NM-NMP treatment. The activity of chitinase in common vetch infected with *C. lentis* or inoculated with *S. tortuosa* was greater, which was consistent with our transcriptome results. These findings indicated that the genes involved in chitinase activity were upregulated, and AM fungi play an important role in regulating the expression of chitinase-related genes and enhancing plant disease resistance. Signal transduction is a key process for plants to defend against pathogens by activating a multi-component defense response (Scheel, 1998). ABA can suppress the necrosis of plant tissue caused by pathogen infection (Ersek et al., 1991). Compared with healthy plants, common vetch infected with *C. lentis* exhibited an upregulation in the genes related to the GO terms “signal transduction” (GO: 0007165), response to pressure (GO: 0006950), and ABA binding (GO: 0010427). The AM fungus may regulate plant multi-component defense responses to enhance plant resistance to pathogens and reduce plant tissue necrosis. This provides additional evidence that the AM fungus reduces plant morbidity and disease index.

The MAPK cascade is an important defense signaling pathway for plants to defend themselves against pathogens (Yoshioka et al., 2009). Plants have a complex network of hormone signals to protect themselves against pathogens (Berens et al., 2017). According to the KEGG pathway analysis, AMP-NMP exhibited upregulation in the genes related to phenylpropanoid biosynthesis (ko00940), plant MAPK signaling pathway (ko04016), plant-pathogen interaction (ko04626), and plant hormone signaling (ko04075) compared with NM-NMP. The upregulation of these genes in AM plants indicates that the AM fungus plays an important role in the response of plants to pathogens. The phenylpropanoid biosynthetic pathway was shown to be involved in the synthesis of secondary metabolites and is a crucial step in plant defense responses (Dixon et al., 2002). The phenylpropanoid biosynthesis pathway occurs by the action of phenylalanine ammonia lyase (PAL), cinnamate 4-hydroxylase (C_4_H), and 4-coumarate: CoA ligase (4CL). PAL not only enhances the defense of plant cell walls but also promotes disease-related proteins such as chitinase (Singh et al., 2016). The products of the 4CL reaction can be used for the synthesis of flavonoids and lignin (Lee et al., 1995), and participate in the defense of plants against pathogens (Zuniga et al., 2019). The AM fungus increased the content of salicylic acid in diseased plants. which will upregulate gene expression in the phenylpropanoid biosynthetic pathway, thus promoting the synthesis of phenylalanine in plants (Zhou et al., 2018). Our results also showed that PAL and 4CL-related genes were more highly expressed in AMP treatments than other treatments, which supports the association between SA and the phenylpropanoid biosynthetic pathway.

In conclusion, this study first reports the transcriptomic characteristics of a new disease of common vetch infected with *C. lentis* and inoculated with *S. tortuosa*. The AM fungus promoted the root growth of common vetch with a changed morphology of plants. They increased the activity and concentrations of plant defensive enzymes, such as chitinase and PPO, increased the concentrations of jasmonic acid and proline, and regulated the expression of genes related to defense against pathogens which reduced the severity of anthracnose in common vetch. The RNA-seq results were consistent with molecular and physiological changes related to plant defense. This study expanded our knowledge of how AM fungi affect the responses of a green manure plant to a pathogen and provides a reference for future studies on common vetch.

## Data Availability Statement

The original contributions presented in the study are publicly available. This data can be found in NCBI, under accession number PRJNA633675.

## Author Contributions

TyD and WZ conceived and designed the experiments. WZ and YL performed the experiments. WZ and TtD analyzed the data. TtD, WZ, YL, and TyD wrote the manuscript. All authors contributed to the article and approved the submitted version.

## Conflict of Interest

The authors declare that the research was conducted in the absence of any commercial or financial relationships that could be construed as a potential conflict of interest.

## Supplementary Material

Supplementary Figure 1 Unigene length distribution.

Supplementary Figure 2 qRT-PCR verification results of differential gene expression. NM = uninoculated with *S. tortuosa*, AM = inoculated with *S. tortuosa*, NMP = NM inoculated with *C. lentis*, and AMP = AM inoculated with *C. lentis*.

Supplementary Table 1 Summary statistics for sequencing for four libraries from NM, AM, NMP, and AMP samples.

Supplementary Table 2 qRT-PCR verification of gene primer information statistics.
