# Supplementary figures and images for: Effect of the AM Fungus Sieverdingia tortuosa on Common Vetch Responses to an Anthracnose Pathogen
Source: Front Microbiol. 2020 Dec 18;11:542623. doi: 10.3389/fmicb.2020.542623 (PMC7775565; doi:10.3389/fmicb.2020.542623)

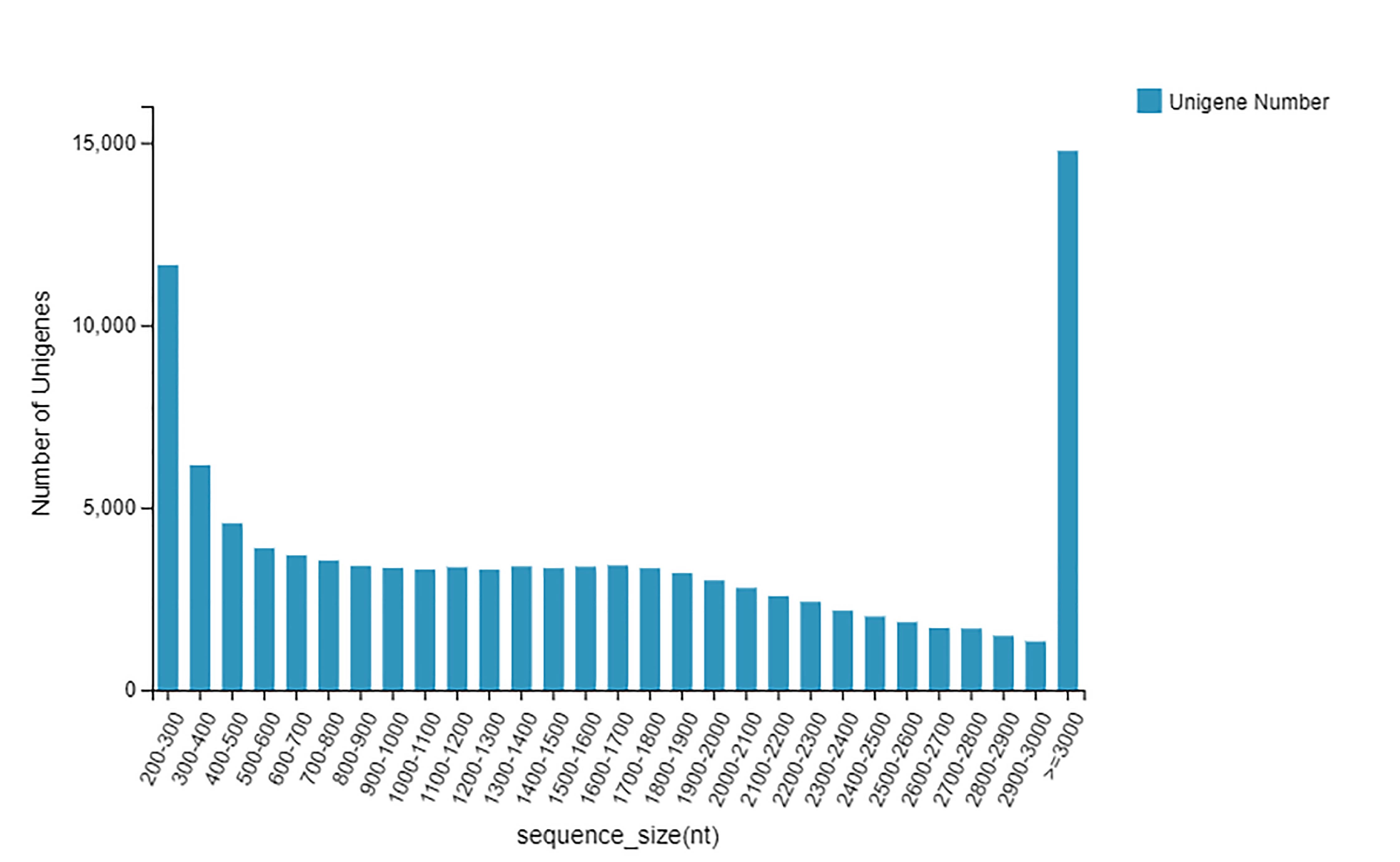

Supplement: Supplementary Figure 1 — Unigene length distribution. [file Image_1.JPEG]

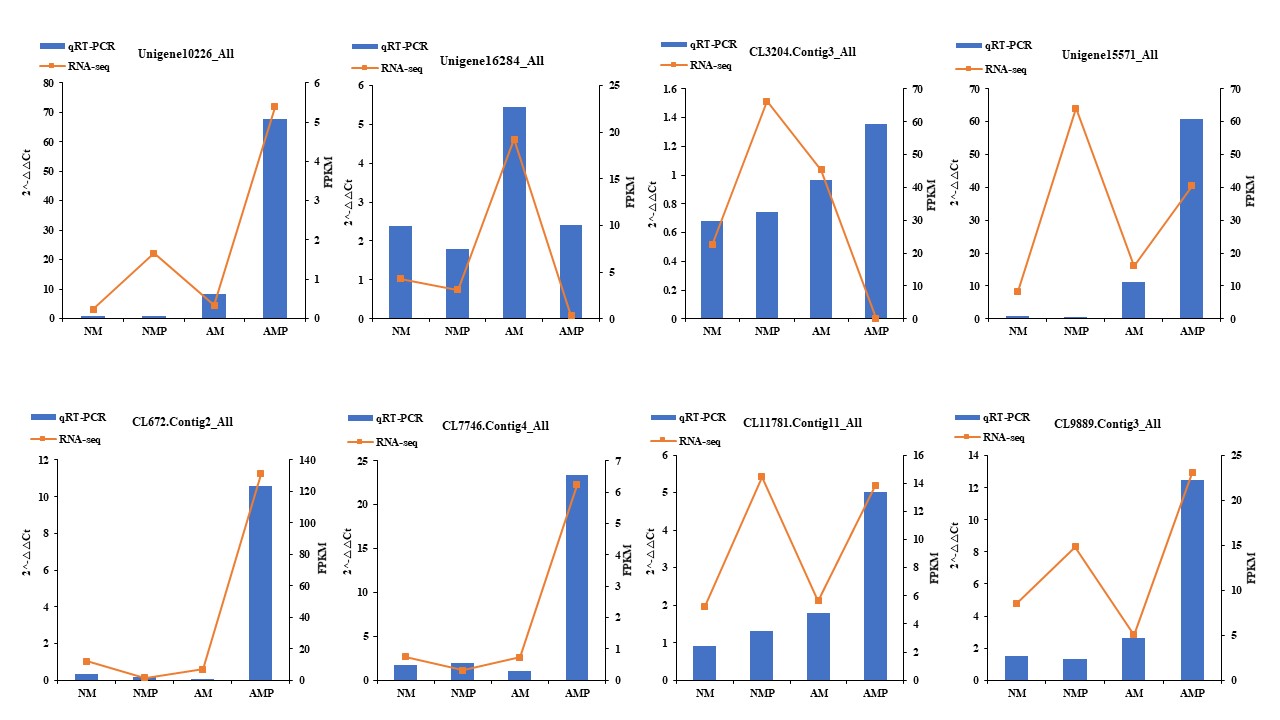

Supplement: Supplementary Figure 2 — qRT-PCR verification results of differential gene expression. NM = uninoculated with S. tortuosa, AM = inoculated with S. tortuosa, NMP = NM inoculated with C. lentis, and AMP = AM inoculated with C. lentis. [file Image_2.JPEG]
