# Supplementary material for: Effect of the AM Fungus Sieverdingia tortuosa on Common Vetch Responses to an Anthracnose Pathogen
Source: Front Microbiol. 2020 Dec 18;11:542623. doi: 10.3389/fmicb.2020.542623 (PMC7775565; doi:10.3389/fmicb.2020.542623)
Supplement: Supplementary Table 1 — Summary statistics for sequencing for four libraries from NM, AM, NMP, and AMP samples. [file Data_sheet_1.docx]

**Supplementary Table 1.** Summary statistics for sequencing for four libraries from NM, AM, NMP and AMP samples

**Supplementary Table 2.** qRT-PCR verification of gene primer information statistics

Supplementary Table 1. Summary statistics for sequencing for four libraries from NM, AM, NMP and AMP samples

| Sample name | Raw reads  (million) | Clean reads  (million) | Clean Reads Ratio (%) | Total Clean Bases (Gb) | GC (%) | Q20 (%) | Q30 (%) | Total Mapped (%) |
| --- | --- | --- | --- | --- | --- | --- | --- | --- |
| NM1 | 87.83 | 75.95 | 86.47 | 11.39 | 39.46 | 97.44 | 90.13 | 85.78 |
| NM2 | 82.56 | 72.19 | 87.44 | 10.83 | 39.52 | 97.24 | 89.34 | 83.97 |
| NM3 | 86.08 | 75.71 | 87.95 | 11.36 | 39.51 | 97.31 | 89.59 | 84.24 |
| AM1 | 86.08 | 74.72 | 86.80 | 11.21 | 39.43 | 97.22 | 89.23 | 84.22 |
| AM2 | 84.32 | 72.62 | 86.13 | 10.89 | 40.66 | 97.25 | 89.34 | 82.99 |
| AM3 | 82.56 | 72.37 | 87.66 | 10.86 | 39.41 | 97.19 | 89.15 | 84.89 |
| NMP1 | 86.07 | 74.05 | 86.03 | 11.11 | 39.31 | 97.33 | 89.95 | 84.27 |
| NMP2 | 86.07 | 74.45 | 86.50 | 11.17 | 39.42 | 97.31 | 89.73 | 85.36 |
| NMP3 | 84.32 | 74.03 | 87.79 | 11.10 | 40.06 | 97.34 | 89.55 | 83.66 |
| AMP1 | 80.81 | 70.60 | 87.37 | 10.59 | 39.26 | 97.20 | 89.11 | 84.04 |
| AMP2 | 86.08 | 73.37 | 85.24 | 11.01 | 39.56 | 97.31 | 89.75 | 85.43 |
| AMP3 | 84.32 | 74.18 | 87.98 | 11.13 | 39.9 | 97.23 | 89.55 | 86.31 |

Note: Q20: percentage of bases with a Phred value ≥20; Q30: percentage of bases with a Phred value ≥30. NM = uninoculated *G. tortuosum*, AM = inoculated with *G. tortuosum*. NMP = NM inoculated with *C. lentis*, AMP = AM inoculated with *C. lentis*. Different lowercase letters at the same columun means there is significant different between treatments at 0.05 level.

Supplementary Table 2. qRT-PCR verification of gene primer information statistics

| Gene ID | primer (5’-3’) | primer (3’-5’) |
| --- | --- | --- |
| Unigene10226_All | TCAAGAAGTGCATCCCAGAA | CCCATAACATCACACCTCCA |
| Unigene16284_All | TGATGCATATTGTGGCACAG | TGGTGTGACAATGTCAGCAA |
| CL3204.Contig3_All | TGGAGCTGCACACAAGGAGC | GCCTTCCTTCTCACCGGTCA |
| Unigene15571_All | TCAAGAAGTGCATCCCAGAA | CCCATAACATCACACCTCCA |
| CL672.Contig2_All | TCTGGTGGAAAACATGGTGG | ATAAGCCCCATGCAAGTGGT |
| CL7746.Contig4_All | TTGTCAACCGATGCCCCTAC | TGAGGTTCCAGGAGCGATGT |
| CL11781.Contig11_All | TCGTTGCGTTCATTGTCTCG | CAAAATGGTTCCCACCCAGA |
| CL9889.Contig3_All | CGTCTGTTCTTGTTTCCGGC | GCGGTCTCGAGCTGTGTCTT |
